# TASKS AND EDUCATION RESOURCES OF ADOLESCENTS WHO ASSIST WITH CAREGIVING FOR A FAMILY MEMBER WITH DEMENTIA

**DOI:** 10.1093/geroni/igz038.1740

**Published:** 2019-11-08

**Authors:** Jennifer Perion, April Ames, Victoria Steiner

**Affiliations:** University of Toledo, Toledo, Ohio, United States

## Abstract

An abundance of research involving adults who care for family members with dementia has guided the creation of supportive programs/services. Much less is known about adolescents who are dementia caregivers. This descriptive secondary analysis utilized data collected during a qualitative examination into the psychological well-being of adolescent dementia caregivers. Eleven adolescent/adult dyads who provided dementia care for a family member completed surveys prior to the adolescents’ participation in focus groups. Five male and six female adolescents ages 12 to 17 and eleven female adults were asked similar questions about caregiving tasks, education resources, and demographic information. Using descriptive statistics, the results of the surveys provide a snapshot of caregiving among a group of adolescents living in northwest Ohio and highlight differences reported by the dyads. Adult accounts of adolescent preparatory education were incongruent with the adolescents’ and did not report their use of books or online caregiving resources. Conversely, three adults (27%), but no adolescents, identified hands-on and observational opportunities as education resources. Adults reported greater adolescent involvement in ten activities of daily living (71%), especially related to bathing, shopping, transportation, and managing medication and finances. Adolescents reported helping with tasks such as eating and laundry more often than adults. While the sample size was small, these findings suggest a need for triangulation when seeking knowledge about adolescent caregiving. These results may inform researchers wishing to investigate the role of adolescent caregivers, as well as guide supportive agencies who provide education materials to families caring for individuals with dementia.

